# Survey data of English teachers' beliefs about second language instruction in Chile

**DOI:** 10.1016/j.dib.2019.104702

**Published:** 2019-10-22

**Authors:** Masatoshi Sato, Juan Carlos Oyanedel

**Affiliations:** Universidad Andres Bello, Chile

**Keywords:** Teachers' beliefs, Apprenticeship of observation, Instructed second language acquisition, Grammar instruction, Corrective feedback, Peer interaction

## Abstract

The data derives from a survey collected from 543 school-level teachers of English in Chile. The survey was originally distributed to 5435 teachers. The survey was developed with an aim of exploring teachers’ beliefs about how second language grammar should be taught. The survey consisted of 50 items in total, in four sections: (a) background information (10 items), (b) beliefs regarding L2 learning and teaching (9 items), (c) beliefs regarding grammar instruction (23 items), and (d) classroom realities (8 items). Except for the background information section, the items took the form of a 6-point Likert scale. The entire dataset is included in an Excel file (.xlsx). The entire questionnaire is included as a supplementary file. The data is connected to the theoretical models proposed in [1]. In [1], those models were proposed based on descriptive statistics (e.g., agreement/disagreement rates) and focus-group interview data. Subsequently, in the current paper, the data was submitted to structural equation modelling to explain the theoretical models. Then, the data is visually depicted with figures created via AMOS.

**Table:** Specifications Table

Subject	Education
Specific subject area	Instructed second language acquisition aims to examine the impact of second language teaching on learners' second language development
Type of data	Microsoft Excel file (.xlsx) Figures (AMOS)
How data were acquired	Survey SurveyMonkey SPSS AMOS
Data format	Raw Figure
Parameters for data collection	The survey targeted (a) school-level teachers, (b) teachers of English, and (c) teachers who reside in Chile.
Description of data collection	The sampling framework was 5435 English teachers registered at the Chilean Ministry of education. With SurveyMonkey, the questionnaire was distributed to those teachers and 543 respondents completed the survey. The data was anonymized throughout the process.
Data source location	Various schools in Chile All 16 regions of Chile
Data accessibility	With the article
Related research article	M. Sato, J.C. Oyanedel, “I think that is a better way to teach but …“: EFL teachers' conflicting beliefs about grammar teaching, System. 84 (2019) 110–122. http://doi.org/10.1016/j.system.2019.06.005.

**Table:** 

**Value of the Data** • The data represents one of the largest samples in the area of second language teachers' beliefs. • The data can be compared across different second language teaching contexts in the world. • The data welcomes different ways of conceptualizing the ways in which second language teachers perceive communicative teaching, grammar instruction, peer interaction, and corrective feedback. • The data offers potential in developing a scale focusing on second language teachers' beliefs about grammar instruction

## 1 Data

**Fig. 1:**
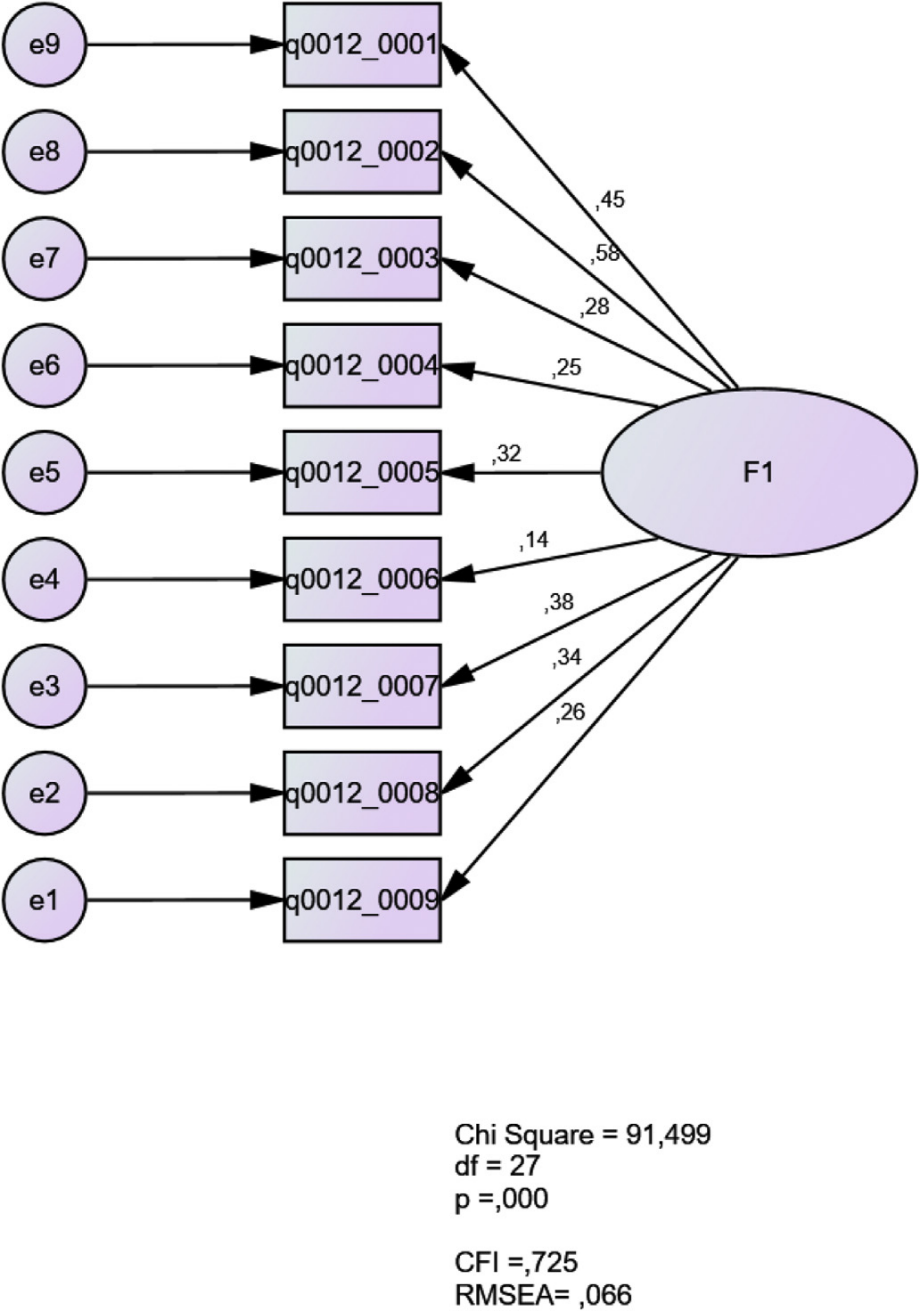
Beliefs of second language acquisition theories.

**Fig. 2:**
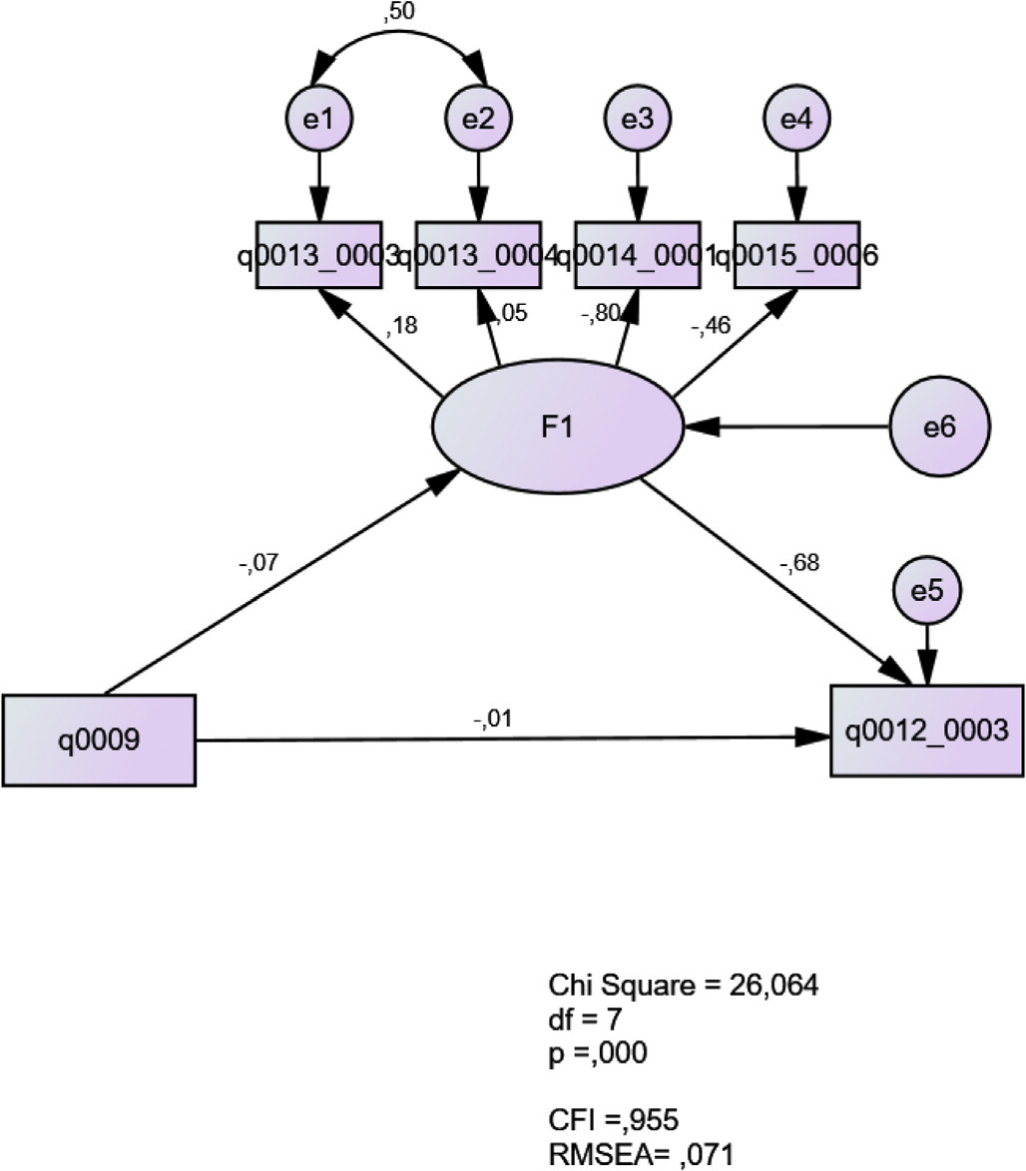
Beliefs about integrated grammar instruction in relation to theoretical beliefs and teaching experience.

**Fig. 3:**
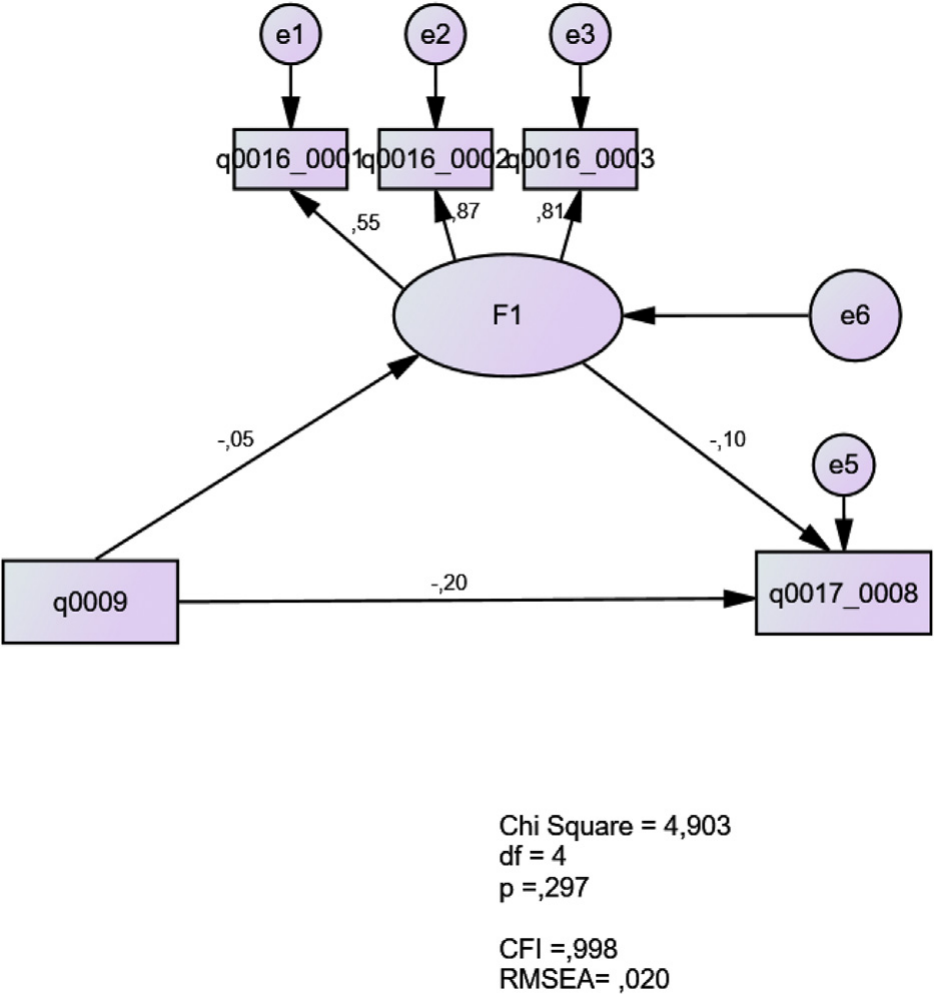
Beliefs about group work in relation to curricular constraints and teaching experience.

**Fig. 4:**
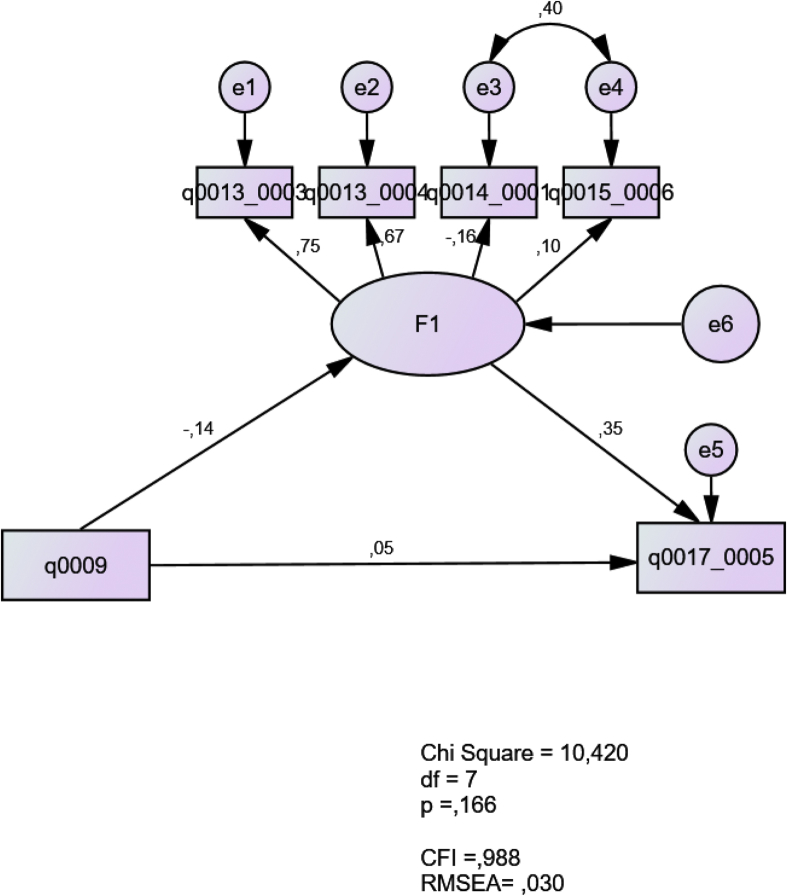
Beliefs about integrated grammar instruction in relation to contextual beliefs and teaching experience.

The data derives from the results of a questionnaire designed to elicit second language teachers’ beliefs regarding communicative language teaching and grammar instruction. The excel sheet (.xlsx) contains raw data from the 50 Likert-scale items. The original questionnaire is also included as a supplementary file. The figures (Fig. 1, Fig. 2, Fig. 3, Fig. 4), based on the confirmatory factor analyses, show the theoretical models suggested in the original article [1]. In Sato & Oyanedel (2019), those models were developed based on (a) descriptive statistics of the questionnaire results, and (b) focus-group interviews.

1. Model 1: Teachers' beliefs about second language acquisition (SLA) theories

In this model, nine items related to the respondents’ understanding of SLA theories (from q0012_0001 to q0012_0009) are tested. A CFA using a maximum likelihood estimation was used. The results show a poor fit with a CFI of 0.725 and a RMSEA of 0.066. The model suggests that the second language teachers held varying beliefs of SLA theories and did not agree among them very much (see Ref. [2]).

2. Model 2: Theoretical conflicts

In this model, the latent construct F1 represents teachers’ beliefs about integrated grammar teaching (q0013_0003; q0013_0004; q0014_0001; q0015_0006). The results show a good fit with a CFI of 0.995 and a RMSEA of 0.071. The construct is negatively predicted by the amount of teaching experience (q0009). The construct negatively predicts the theoretical belief in the importance of explicit understanding of grammatical rules (q0012_0003). In other words, the model suggests that (a) the more experienced teachers thought that explicit understanding of grammatical rules is less necessary, and (b) the beliefs of integrated grammar teaching had an inverse relationship with those of explicit grammar teaching (see Ref. [3]).

3. Model 3: Experiential conflicts

In this model, the latent construct F1 represents teachers' beliefs about group work (q0016_0001; q0016_0002; q0016_0003). The results show a good fit with a CFI of 0.998 and a RMSEA of 0.020. The teaching experience (q0009) negatively predicts the latent construct. The construct negatively predicts the beliefs regarding curriculum constraints to implement group work (q0012_0003). The model suggests that as the teachers became more experienced, they became less reliant on and favourable of group work; however, those teachers felt less constrained by the curriculum to implement group work. Also, it suggests that teachers’ favourable view of group work suppresses their feelings of being constrained by the curriculum (see Ref. [4]).

4. Model 4: Contextual conflicts

In this model, the latent construct F1 represents teachers’ beliefs about integrated grammar teaching (q0013_0003; q0013_0004; q0014_0001; q0015_0006). The results show a good fit with a CFI of 0.988 and a RMSEA of 0.030. The teaching experience (q0009) positively predicts the belief of the appropriateness of communicative teaching to the Chilean context (q0017_0005). The latent construct positively predicts the contextual beliefs. The model suggests that the more experience teachers had, the more they thought that communicative teaching was appropriate for the Chilean context (see Ref. [5]). Their positive perceptions of integrated grammar teaching led to their feeling that the type of instruction was appropriate for the Chilean context.

## 2 Experimental design, materials, and methods

The survey, implemented via SurveyMonkey, was distributed via email to 5435 English teachers throughout Chile, with the help of Inglés Abre Puertas (English Opens Doors), a governmental program of the Ministry of Education of Chile aiming to promote the teaching of English. The survey was administered in August and September in 2014. Data was collected with pre-coded alternatives. No imputation procedures were used. AMOS’ estimation of means and intercepts were used to account for missing data.
