# Supplementary material for: Survey data of English teachers' beliefs about second language instruction in Chile
Source: Data Brief. 2019 Oct 22;27:104702. doi: 10.1016/j.dib.2019.104702 (PMC6838429; doi:10.1016/j.dib.2019.104702)
Supplement: Multimedia component 2 [file mmc2.docx]

*Note*. The questionnaire was distributed using online software (SurveyMonkey). Hence, the outlook of the questionnaire differs from that the respondents actually saw. The section headings were not included in the actual questionnaire.

| **SD** | **D** | **MD** | **MA** | **A** | **SA** |
| --- | --- | --- | --- | --- | --- |
| **Strongly disagree** | **Disagree** | **Mildly**  **disagree** | **Mildly**  **agree** | **Agree** | **Strongly**  **agree** |

**Section A: Background information**

| 1 | Is English your mother tongue? | __________ Yes __________ No |
| --- | --- | --- |
| 2  (q0003) | Which age group do you belong to? | 20-29 / 30-39 / 40-49 / 50-59 / 60-69 / more than 70 |
| 3  (q0004) | For how many years have you studied English in formal settings (e.g., schools, universities, language schools)? | less than 5 / 5-6 / 7-8 / 9-10 / 11-12 / more than 13 |
| 4  (q0005) | I learned English mainly via grammar-focused, teacher-centered lessons and vocabulary memorization. | SD — D — MD — MA — A — SA |
| 5  (q0006) | Have you ever taken a course focusing on second language learning theories and/or Applied Linguistics? | __________ Yes / __________ No |
| 6  (q0007) | Have you completed a training program specifically for English teaching? | __________ Yes / __________ No |
| 7  (q0008) | Do you have a graduate-level degree and/or certificate of education, applied linguistics, or TESOL? | __________ Yes / __________ No |
| 8  (q0009) | For how many years have you worked as a full-time (more than 20 hours/week) English teacher? | Less than 2 / 2-4 / 4-7 / 7-10 / 10-13 / more than 13 |
| 9  (q0010) | The age group (grades) that I currently teach (mainly) is: | 1-2 / 3-4 / 5-6 / 7-8 / 1°-2° *Medio* / 3°-4° *Medio* |
| 10  (q0011) | The region where I teach is: | Arica and Parinacota / Tarapacá / Antofagasta / Atacama / Coquimbo / Valparaíso / Metropolitana / O’Higgins / Maule / Bibío / Araucanía / Los Ríos / Los Lagos / Aysén / Magallanes |

**Section B: Beliefs regarding second language learning and teaching**

| 11  (q0012  _0001) | Learners can become good second language users simply by being exposed to the language. | SD — D — MD — MA — A — SA |
| --- | --- | --- |
| 12  (q0012  _0002) | Second languages are learned mainly through imitation. | SD — D — MD — MA — A — SA |
| 13  (q0012  _0003) | Learners need to achieve explicit understanding of grammar rules to learn a second language. | SD — D — MD — MA — A — SA |
| 14  (q0012  _0004) | The earlier a second language is introduced in school programs, the greater the likelihood of success in learning. | SD — D — MD — MA — A — SA |
| 15  (q0012  _0005) | Classrooms are good places to learn about language but not for learning how to use language. | SD — D — MD — MA — A — SA |
| 16  (q0012  _0006) | A teacher who learned English as a second language can be a better English teacher than a native speaker of English. | SD — D — MD — MA — A — SA |
| 17  (q0012  _0007) | The way a teacher was taught English affects the way he/she teaches English. | SD — D — MD — MA — A — SA |
| 18  (q0012  _0008) | The more teaching experience a teacher has, the better he is at teaching English. | SD — D — MD — MA — A — SA |
| 19  (q0012  _0009) | An understanding of theories of second language acquisition helps second language teachers teach better. | SD — D — MD — MA — A — SA |

**Section C: Beliefs regarding grammar instruction**

**C-1: Communicativeness**

| 20  (q0013  _0001) | A second language classroom should always be communicative. | SD — D — MD — MA — A — SA |
| --- | --- | --- |
| 21  (q0013  _0002) | It is important to practice a second language in situations simulating real life. | SD — D — MD — MA — A — SA |
| 22  (q0013  _0003) | Grammar rules can be learned through communicative activities. | SD — D — MD — MA — A — SA |
| 23  (q0013  _0004) | It is useful for learners to comprehend grammar rules through their own discovery. | SD — D — MD — MA — A — SA |
| 24  (q0013  _0005) | Students should be active participants in the classroom. | SD — D — MD — MA — A — SA |

**C-2: Grammar instruction**

| 25  (q0014  _0001) | Grammatical rules should be taught explicitly (e.g., explanations of different types of conditional sentences). | SD — D — MD — MA — A — SA |
| --- | --- | --- |
| 26  (q0014  _0002) | It is important to learn grammar in order to speak accurately in a second language (e.g., English). | SD — D — MD — MA — A — SA |
| 27  (q0014  _0003) | It is important to learn grammar in order to speak fluently in a second language (e.g., English). | SD — D — MD — MA — A — SA |
| 28  (q0014  _0004) | Grammar drills (e.g., conjugation exercises; fill-in-the-gap activities) are important for learning a second language. | SD — D — MD — MA — A — SA |

**C-3: Corrective feedback**

| 29  (q0015  _0001) | Grammatical errors are evidence of second language development. | SD — D — MD — MA — A — SA |
| --- | --- | --- |
| 30  (q0015  _0002) | Learners’ grammatical errors when speaking in a second language must always be corrected. | SD — D — MD — MA — A — SA |
| 31  (q0015  _0003) | Learners’ grammatical errors should be corrected as soon as they are made. | SD — D — MD — MA — A — SA |
| 32  (q0015  _0004) | Teachers should correct learners’ grammatical errors by giving them the correct answer. | SD — D — MD — MA — A — SA |
| 33  (q0015  _0005) | Teachers should point out learners’ grammatical errors without giving them the correct answer. | SD — D — MD — MA — A — SA |
| 34  (q0015  _0006) | When correcting learners’ grammatical errors, teachers should be explicit so that the learners notice the correction. | SD — D — MD — MA — A — SA |
| 35  (q0015  _0007) | Correcting a learner when he/she is talking interrupts the flow of communication. | SD — D — MD — MA — A — SA |
| 36  (q0015  _0008) | Communication should be stopped when the teacher corrects learners’ errors. | SD — D — MD — MA — A — SA |
| 37  (q0015  _0009) | Correcting a learner in front of his/her classmates may embarrass him/her. | SD — D — MD — MA — A — SA |

**C-4: Group work**

| 38  (q0016  _0001) | Group activities are useful to get students involved in class. | SD — D — MD — MA — A — SA |
| --- | --- | --- |
| 39  (q0016  _0002) | Students have more chance to practice speaking during group work than during teacher-centered activities. | SD — D — MD — MA — A — SA |
| 40  (q0016  _0003) | Students speak more freely during group work than during teacher-centered activities. | SD — D — MD — MA — A — SA |
| 41  (q0016  _0004) | Students tend to learn mistakes from each other when working in pairs or groups. | SD — D — MD — MA — A — SA |
| 42  (q0016  _0005) | Students should be encouraged to correct each other’s grammatical errors during group work. | SD — D — MD — MA — A — SA |

**Section D: Classroom realities**

| 43  (q0017  _0001) | The size of my class prohibits me from giving communicative classes. | SD — D — MD — MA — A — SA |
| --- | --- | --- |
| 44  (q0017  _0002) | My students believe that communicative activities are just for fun but not for learning. | SD — D — MD — MA — A — SA |
| 45  (q0017  _0003) | My students want me to teach them grammar explicitly. | SD — D — MD — MA — A — SA |
| 46  (q0017  _0004) | My students are waiting for me to correct their grammatical errors when they are speaking. | SD — D — MD — MA — A — SA |
| 47  (q0017  _0005) | Communicative teaching is appropriate for the Chilean culture. | SD — D — MD — MA — A — SA |
| 48  (q0017  _0006) | My students want me to teach the second language (English) in their first language (Spanish). | SD — D — MD — MA — A — SA |
| 49  (q0017  _0007) | Teacher-centered classes are easier for the teacher to give than student-centered classes. | SD — D — MD — MA — A — SA |
| 50  (q0017  _0008) | The national/school curriculum prevents me from giving communicative classes. | SD — D — MD — MA — A — SA |

**Section E: Well-being**

0 = “No satisfaction at all” ——— 10 = “Completely satisfied”

1. Thinking about your own life and personal circumstances, how satisfied are you with your life as a whole?

How satisfied are you with…?

1. your standard of living? [Standard of Living]
2. your health? [Personal Health]
3. what you are achieving in life? [Achieving in Life]
4. your personal relationships? [Personal Relationships]
5. how safe you feel? [Personal Safety]
6. feeling part of your community? [Community-Connectedness]
7. your future security? [Future Security]
8. your spirituality or religion? (Spirituality or Religion)
